# Association between hs-CRP and depressive symptoms: a cross-sectional study

**DOI:** 10.3389/fpsyt.2024.1339208

**Published:** 2024-03-26

**Authors:** Yewei Ji, Jinmin Wang, Huaqin Chen, Jiawen Li, Mingyang Chen

**Affiliations:** Department of Internal Neurology, The Second People’s Hospital Affiliated to Fujian University of Traditional Chinese Medicine, Fuzhou, China

**Keywords:** NHANES, cross-sectional study, high-sensitivity C-reactive protein, depressive symptoms, inflammation

## Abstract

**Background and aim:**

High-sensitivity C-reactive protein (hs-CRP) is a sensitive measure of low-grade inflammation and appears superior to conventional blood tests in assessing cardiovascular disease. The purpose of this investigation was to explore the link between high-sensitivity CRP and depressive symptoms among adults.

**Methods and results:**

Multiple logistic regression and smoothed curve fitting were used to investigate the association between hs-CRP and depressive symptoms based on data from the, 2017-2020 National Health and Nutrition Examination Survey (NHANES). Subgroup analyses and interaction tests were used to assess the stability of this relationship across populations. The study comprised 6,293 non-clinical participants, which included 549 individuals with depressive symptoms. The prevalence of depressive symptoms was found to increase with increasing levels of hs-CRP. This trend persisted even after quartetting hs-CRP levels. In the fully adjusted model, each unit increase in hs-CRP was associated with a 10% increase in the odds of depressive symptoms (OR=1.10,95%CI:1.01-1.21). Participants in the highest quartile of hs-CRP had a 39% higher prevalence of depressive symptoms compared to those in the lowest quartile (OR=1.39,95%CI:1.01-1.92). Additionally, this positive correlation was more pronounced in men.

**Conclusions:**

In adult Americans, there exists a positive association between elevated hs-CRP levels and depressive symptoms, with a more prominent manifestation of this association observed in males.

## 1 Introduction

Depression, a common mental disorder, is gradually increasing in prevalence worldwide, posing a significant threat to individual and societal well-being (1). By, 2030, depression is predicted to become the leading cause of disability (2). The pathogenesis of depression involves multiple risk factors, including genetic, psychological, biological, and environmental factors. Current research highlights that depression may be associated with neuroinflammation, but the underlying mechanisms remain unclear. Therefore, it is essential to study the correlation between depression and inflammation.

C-reactive protein, a marker of the acute-phase inflammatory response, has been widely used to measure low-level inflammation in psychiatric disorders and has provided valuable information in cardiovascular disease diagnosis and risk prediction (3). Compared to CRP, hs-CRP is more commonly used to study low-level inflammation and predict the risk of developing inflammatory diseases. It is also a sensitive indicator of low-level immune inflammation. There is growing evidence that there is an association between depression and inflammation and that inflammation may be an essential pathological factor, not just an outcome (4). Poor efficacy of antidepressants has been reported to be linked to severe immune activation. Moreover, anti-inflammatory treatments display antidepressive effects and are beneficial for patients suffering from depression (5–7). Several ongoing randomized controlled trials are currently recruiting depressed patients with elevated CRP levels for the study of anti-inflammatory treatments for depression (8). Research suggests that evaluating inflammatory markers can help to develop better prevention and treatment strategies for depression (9). In this context, hs-CRP may be valuable in predicting and assessing early depression as an especially sensitive acute inflammation marker.

Epidemiological evidence on the association between hs-CRP and depression in the general population has been inconsistent. Some studies have found an association between depression and elevated hs-CRP levels (10–13), while others have not (14, 15). Furthermore, variations in the association between depression and hs-CRP levels have been observed in studies conducted in diverse populations, including differences related to gender, age, race, or other potential confounders.

Therefore, we used data from the, 2017-2020 National Health and Nutrition Examination Survey (NHANES) to investigate the association between serum hs-CRP levels and the likelihood of incident depressive symptoms in US adults.

## 2 Materials and methods

### 2.1 Study population

The Centers for Disease Control and Prevention conducts a nationally representative survey called the NHANES (16). The Research Ethics Review Board of the National Centre for Health Statistics (NCHS) approved the study procedure. All participants provided written consent at the time of recruitment (17). All NHANES data are publicly available at https://www.cdc.gov/nchs/nhanes/.

**Figure 1:**
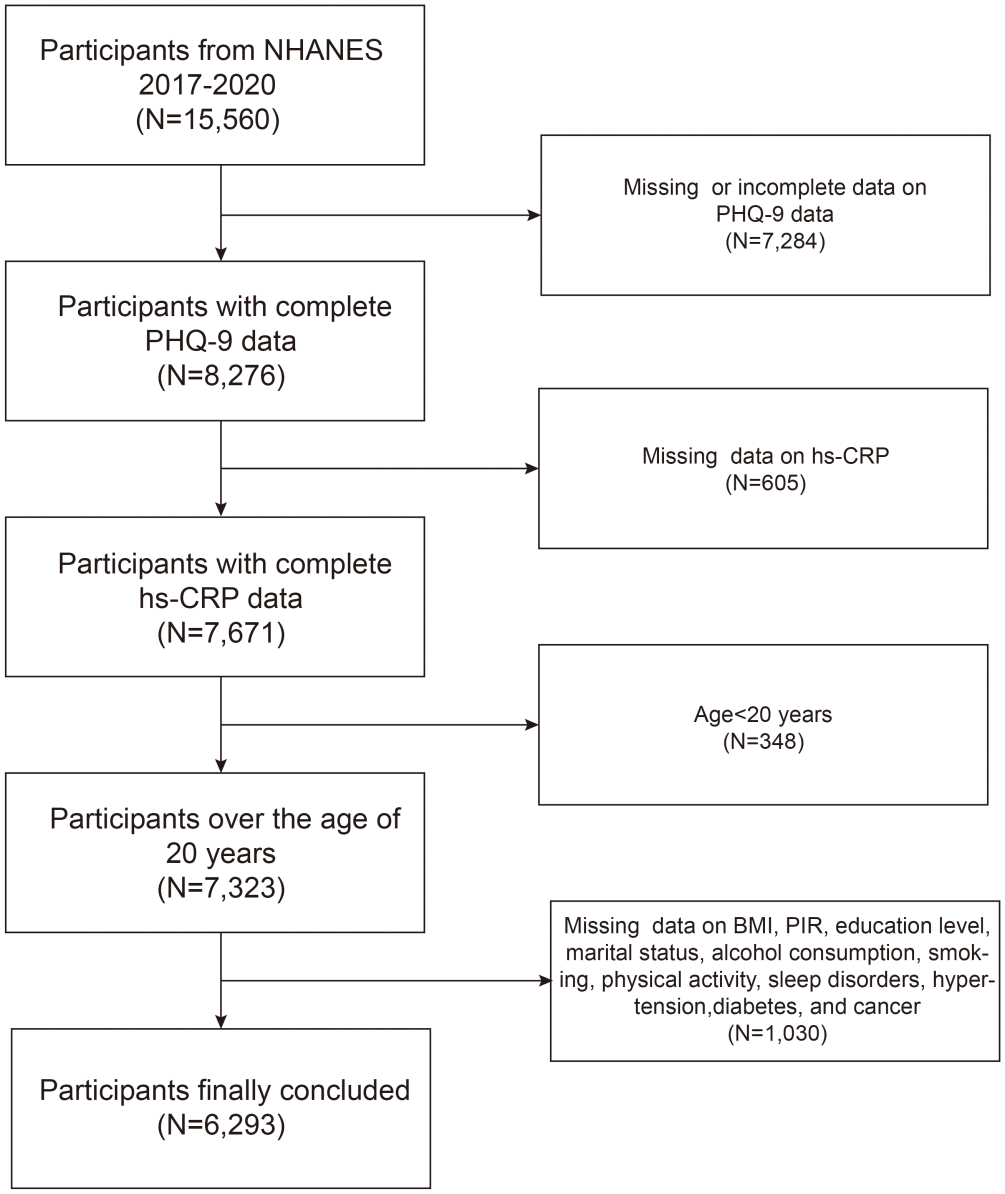
Flowchart of participants inclusion and exclusion.

Our study was conducted using prepandemic NHANES data from, 2017 to, 2020. At the outset, there were 15,560 participants in the cohort. However, after excluding cases with incomplete or missing PHQ-9 data (n=7,284), those without available hs-CRP level data (n=605), those aged <20 years (n=348), and those with missing information on other covariates (n=1,030), the final number of participants was reduced to 6,293, which is shown in **Figure 1**.

### 2.2 Exposure and outcome definition

The exposure variable in this study is hs-CRP, an acute-phase protein synthesized in the liver. CRP is synthesized in the liver as an acute-phase protein and is involved in complement activation, phagocytosis enhancement, and detoxification of substances released from damaged tissue. Although it is one of the most sensitive indicators of inflammation, CRP levels are nonspecific. They may increase within six hours of an inflammatory stimulus. Hs-CRP testing refers to the laboratory’s ultra-sensitive detection technology to accurately measure small CRP concentrations. It is a highly sensitive indicator for detecting mild inflammatory conditions at low levels.

The NHANES employed the validated nine-item Public Health Questionnaire (PHQ-9) to measure depressive symptoms. The well-known survey offers a sensitivity and specificity of 88% (18). The PHQ-9 is a highly effective tool for screening depressive symptoms, which was administered to evaluate depressive symptoms over the past two weeks. Technical term abbreviations are explained when first used. It measures depressive symptoms in participants over the previous two weeks, including those experiencing restless sleep, loss of appetite, and loneliness. Each item is evaluated on a 4-point ordinal scale indicating the frequency of the symptom (0, not at all; 1, several days; 2, more than half the days; 3, nearly every day) (19). The PHQ-9 score is the sum of the scores from the nine items. Participants with a score of 10 or more were defined as having symptoms of depression. Both the prevalence of depressive symptoms occurrence and the PHQ-9 total score are designed as outcome variables in this study.

### 2.3 Covariates

The covariates include age (years), gender (male/female), race (Mexican American/other Hispanic/non-Hispanic White/non-Hispanic Black/other races), alcohol consumption(yes/no), smoking status (yes/no), physical activity (yes/no), sleep disorders (yes/no), education level (less than high school/high school or general educational development/above high school), family income to poverty ratio (PIR), marital status(yes/no) and body mass index (BMI), hypertension, diabetes, coronary heart disease, and cancer(Doctor informed you had any condition yes vs no). PIR is a ratio of self-reported income to the local poverty level. Participants were considered smokers if they had smoked ≥100 cigarettes in their life. The disease history was defined as a self-reported physician diagnosis of hypertension, diabetes, coronary heart disease, and cancer. You can find explanations for all variables on the official NHANES website (https://www.cdc.gov/nchs/nhanes).

### 2.4 Statistical analysis

All statistical analyses followed Centers for Disease Control and Prevention guidelines, with the appropriate NHANES sampling weight applied and adjusted for the complex multistage cluster survey design during analysis.

Differences between depressed and non-depressed subjects were assessed using the Kruskal-Wallis rank sum test (for continuous variables) or the chi-squared test (for categorical variables). Multivariate logistic regression models explored the independent relationships between hs-CRP levels and depressive symptoms and the PHQ-9 total score across different model specifications. After hs-CRP levels were transformed from a continuous variable to a categorical variable (quartile), trend tests were used to examine linear trends in the association between hs-CRP and depressive symptoms. No covariate adjustments were made in Model 1. Model 2 was adjusted for age, gender, and race. Model 3 involved adjustments for age, gender, race, PIR, BMI, education level, marital status, alcohol intake, smoking status, hypertension, diabetes, coronary heart disease, and cancer. Subgroup analyses were conducted to examine the association between hs-CRP and depressive symptoms in different age, gender, BMI, hypertension, diabetes, coronary heart disease, and cancer subgroups, with interaction tests to investigate the consistency of these associations across subgroups. Smoothed curve fitting was used to investigate the nonlinearity of the association between hs-CRP and depressive symptoms. All analyses were performed with R (version 4.3.1) or Empowerstats (version 2.0). Statistical significance is defined as two-sided p < 0.05.

## 3 Results

### 3.1 Baseline characteristics of participants

The study included 6,293 participants aged 20 years and above, with 48.59% male and 51.41% female. The median age of all participants was 52 years (25th, 75th percentiles: 36, 64 years), with a median BMI of 28.90 kg/m^2^ (25th, 75th percentiles: 25.00, 33.90 kg/m^2^). The median hs-CRP level was 2.00 mg/L (25th, 75th percentiles: 0.86, 4.47 mg/L), and the median total PHQ-9 score was 2.00 (25th, 75th percentiles: 0.00, 5.00). Among these participants, 42.62% were smokers, 91.72% were alcohol users, 58.97% were married or living with a partner, 59.65% had at least a high school education, 29.80% had self-reported sleep disorders, 41.46% were physically active, and the diagnoses of hypertension, diabetes mellitus, coronary heart disease, and cancer were 38.09%, 15.24%, 4.53%, and 10.61%, respectively.

Among all participants, individuals with depressive symptoms were more likely to be female (61.75%), smokers (54.64%), and drinkers (94.55%). They were also more likely to have a high school education or higher (48.45%) and to be married or cohabiting with a partner (45.72%). Additionally, they were less physically active (69%). The study found that individuals with depressive symptoms were more likely to have a self-reported sleep disorder (66.12%) and less likely to have hypertension (49.54%), diabetes mellitus (21.68%), coronary heart disease (6.24%), and cancer (13.11%). All differences were statistically significant (p<0.05). Patients with depressive symptoms also had significantly higher BMI and serum hs-CRP levels compared to those without depressive symptoms (**Table 1**). In addition, **Supplementary Table 1** demonstrates the general demographic characteristics of the participants excluded and included in this study.

**Table 1:** Basic characteristics of participants. (N=6,293).

Characteristics	Total	Non- depressive symptoms	Depressive symptoms	*P*-value*
	N=6,293	N=5,744	N=549	
Age,(year)	52.00 (36.00-64.00)	52.00 (36.00-64.00)	51.00 (36.00-62.00)	0.046
Gender,(%)				<0.001
Male	48.59	49.58	38.25	
Female	51.41	50.42	61.75	
Race,(%)				0.135
Mexican American	11.54	11.61	10.75	
Other Hispanic	9.82	9.58	12.39	
Non-Hispanic White	37.84	37.66	39.71	
Non-Hispanic Black	24.69	24.86	22.95	
Other Races	16.11	16.30	14.21	
Education level,(%)				<0.001
Less than high school	16.49	15.86	23.13	
High school or GED	23.85	23.42	28.42	
Above high school	59.65	60.72	48.45	
Smoking,(%)				<0.001
Yes	42.62	41.47	54.64	
No	57.38	58.53	45.36	
Alcohol consumption,(%)				0.012
Yes	91.72	91.45	94.55	
No	8.28	8.55	5.45	
Marital status,(%)				<0.001
Married/Living with Partner	58.97	60.24	45.72	
Widowed/Divorced/Separated	22.34	21.57	30.42	
Never married	18.69	18.19	23.86	
Physical activity,(%)				<0.001
Yes	41.46	42.51	30.42	
No	58.54	57.49	69.58	
Hypertension,(%)				<0.001
Yes	38.09	37.00	49.54	
No	61.91	63.00	50.46	
Diabetes,(%)				<0.001
Yes	15.24	14.62	21.68	
No	81.82	82.38	75.96	
Borderline	2.94	2.99	2.37	
Cancer,(%)				0.047
Yes	10.61	10.38	13.11	
No	89.39	89.62	86.89	
Coronary heart disease,(%)				0.044
Yes	4.53	4.36	6.24	
No	95.47	95.64	93.76	
Sleep disorders,(%)				<0.001
Yes	29.80	26.32	66.12	
No	70.20	73.68	33.88	
BMI,(kg/m^2^)	28.90 (25.00-33.90)	28.70 (24.90-33.60)	31.00 (25.90-36.40)	<0.001
PIR	2.32 (1.21-4.31)	2.41 (1.24-4.45)	1.51 (0.82-2.72)	<0.001
hs-CRP level,(mg/L)	2.00 (0.86-4.47)	1.92 (0.83-4.39)	2.99 (1.22-5.74)	<0.001
PHQ-9 score	2.00 (0.00-5.00)	1.00 (0.00-4.00)	13.00 (11.00-16.00)	<0.001

Median (Q1-Q3) for continuous variables, (%)for categorical variables.

PIR, family income to poverty ratio; BMI, body mass index; PHQ, Patient Health Questionnaire; hs-crp, high-sensitivity C-reactive protein.

### 3.2 Association between hs-CRP and depressive symptoms

**Figure 2:**
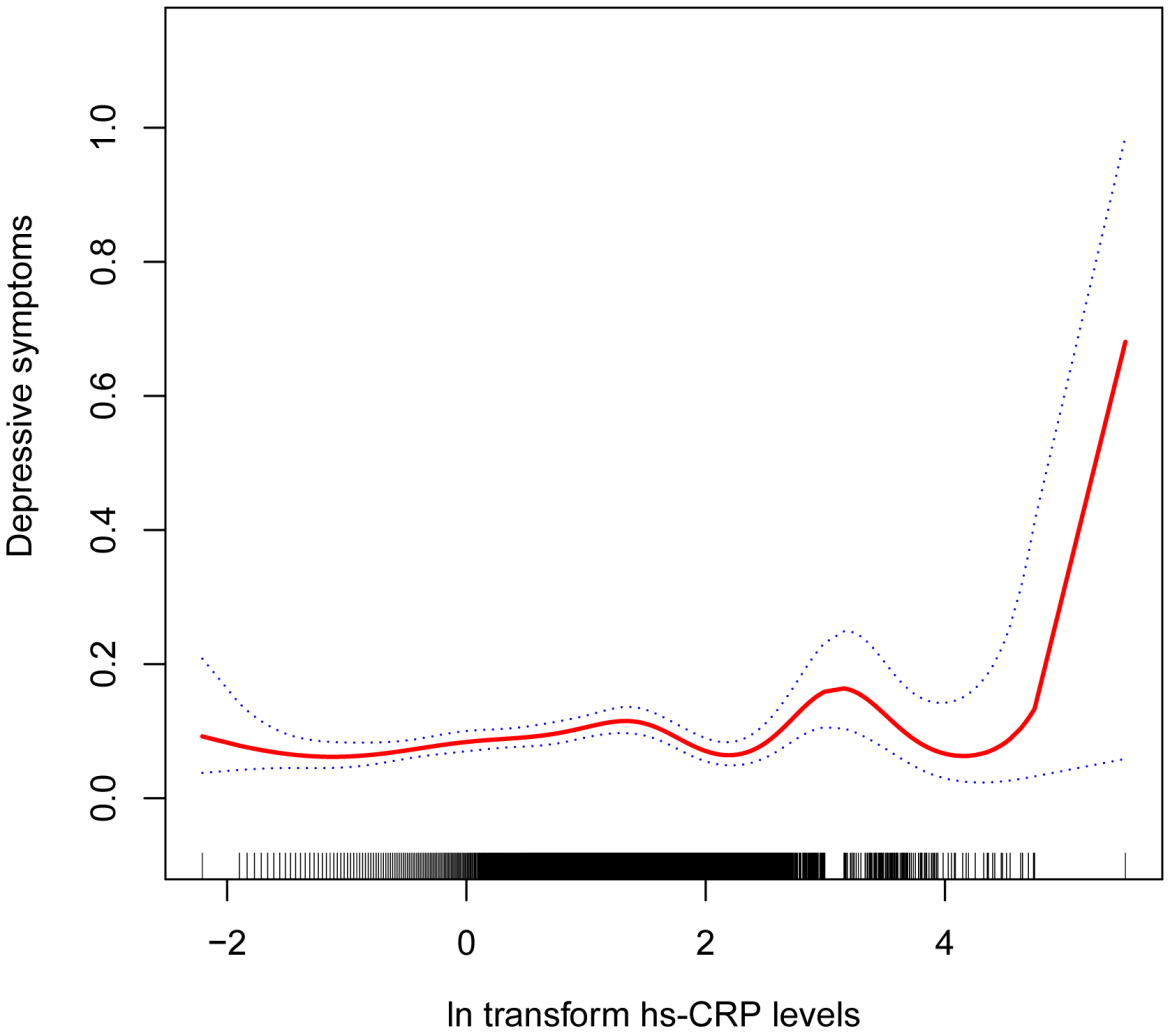
Non-linear correlation between hs-CRP and depressive symptoms. The red line denotes the curve fit for the variables, while the blue bands indicate the 95% confidence interval.

**Figure 3:**
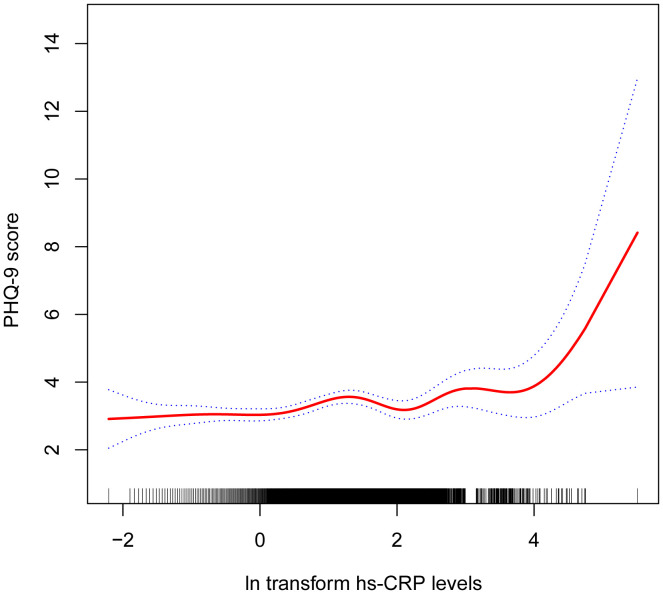
Non-linear correlation between hs-CRP and PHQ-9 score. The red line denotes the curve fit for the variables, while the blue bands indicate the 95% confidence interval.

**Table 2** shows the association between hs-CRP and depressive symptoms. Given the highly skewed distribution of hs-CRP levels, we examined the linear relationship between the log-transformed values of hs-CRP and depressive symptoms. The results indicated a significant positive correlation between hs-CRP and depressive symptoms, as well as PHQ-9 scores. This positive association remained robust in the fully adjusted model (Model 3). Each 1-unit increase in hs-CRP was associated with a 10% increased probability of depressive symptoms (OR=1.10,95%CI=1.01-1.21) and a 0.17 rise in PHQ-9 score (β=0.17,95%CI=0.07-0.26). Even after dividing hs-CRP into quartiles, the previously mentioned links remained statistically significant (P for trend <0.05). Those individuals in the highest hs-CRP quartile displayed a 39% increased prevalence of depressive symptoms (OR=1.39,95%CI=1.01-1.92) compared to those in the lowest quartile. Moreover, the smoothed curve fitting findings demonstrate a non-linear inverse relationship between hs-CRP, depressive symptoms, and PHQ-9 score (**Figures 2**, **3**).

**Table 2:** Association between in transform hs-CRP level and PHQ-9 score and depressive symptoms.

ln transform hs-CRP level	PHQ-9 Score	Depressive symptoms
	β (95% CI)	OR (95% CI)
Crude model (Model 1)		
Continuous	0.46 (0.37, 0.55)	1.29 (1.20, 1.39)
Categories		
Quartile 1	0 (Ref)	1 (Ref)
Quartile 2	0.33 (0.04, 0.63)	1.54 (1.15, 2.05)
Quartile 3	1.07 (0.78, 1.36)	2.31 (1.76, 3.04)
Quartile 4	1.26 (0.97, 1.55)	2.22 (1.69, 2.92)
P for trend	<0.001	<0.001
Minimally adjusted model (Model2)		
Continuous	0.42 (0.33, 0.51)	1.27 (1.17, 1.37)
Categories		
Quartile 1	0 (Ref)	1 (Ref)
Quartile 2	0.35 (0.06, 0.64)	1.56 (1.17, 2.09)
Quartile 3	1.02 (0.73, 1.31)	2.29 (1.74, 3.02)
Quartile 4	1.13 (0.84, 1.43)	2.11 (1.59, 2.79)
P for trend	<0.001	<0.001
Fully adjusted model (Model3)		
Continuous	0.17 (0.07, 0.26)	1.10 (1.01, 1.21)
Categories		
Quartile 1	0 (Ref)	1 (Ref)
Quartile 2	0.15 (-0.12, 0.42)	1.41 (1.04, 1.92)
Quartile 3	0.51 (0.23, 0.79)	1.77 (1.31, 2.39)
Quartile 4	0.40 (0.10, 0.70)	1.39 (1.01, 1.92)
P for trend	0.002	0.044

Model 1: no covariates were adjusted.

Model 2: age, gender, and race were adjusted.

Model 3: age, gender, race, PIR, BMI, education level, alcohol consumption, smoking, physical activity, sleep disorders, diabetes, hypertension, cancer, and coronary heart disease were adjusted.

PIR, family income to poverty ratio; BMI, body mass index; PHQ, Patient Health Questionnaire; hs-crp, high-sensitivity C-reactive protein.

### 3.3 Subgroup analyses

Although the relationship between heightened serum hs-CRP levels and depressive symptoms and PHQ-9 score was generally positively correlated in most subgroups, this linear positive correlation varied among some subgroups (P for interaction < 0.05). In men, each 1-unit increase in hs-CRP was associated with a 0.29 increase in PHQ-9 score (β=0.29, 95% CI=0.15-0.42) and a 21% higher likelihood of experiencing depressive symptoms (OR=1.21, 95% CI=1.05-1.40). However, among women, the correlation between hs-CRP and depressive symptoms and PHQ-9 scores became non-significant (β=0.04, 95% CI=-0.10-0.17; OR=1.00, 95% CI=0.88, 1.13). Additionally, the positive correlation between hs-CRP and PHQ-9 was statistically significant in participants aged 60 years and older (β=0.30, 95% CI=0.15-0.45). Moreover, each unit increase in hs-CRP was associated with a 0.54 increase in PHQ-9 score (β=0.68, 95% CI=0.26-1.11) in participants with coronary artery disease than in those without coronary artery disease (β=0.14, 95% CI=0.04-0.23). Importantly, interaction tests showed no significant dependence of age, BMI, marital status, hypertension, diabetes, coronary heart disease, and cancer on the positive association of hs-CRP with depressive symptoms (**Tables 3**, **4**).

**Table 3:** Subgroup analysis of the association between ln transform hs-CRP level and Depressive symptoms.

Subgroup	OR (95% CI)	*P* for interaction
Age		0.167
<60years	1.03 (0.92, 1.16)	
≥60 years	1.18 (1.01, 1.37)	
Gender		0.046
Male	1.21 (1.05, 1.40)	
Female	1.00 (0.88, 1.13)	
BMI		0.371
<25 kg/m^2^	1.08 (0.90, 1.30)	
25-29.9 kg/m^2^	1.24 (1.04, 1.48)	
≥30 kg/m^2^	1.06 (0.93, 1.21)	
Marital status,(%)		0.072
Married/Living with Partner	1.22 (1.07, 1.40)	
Widowed/Divorced/Separated	1.04 (0.88, 1.24)	
Never married	0.94 (0.77, 1.14)	
Hypertension(%)		0.816
Yes	1.09 (0.95, 1.25)	
No	1.11 (0.98, 1.26)	
Diabetes(%)		0.184
Yes	1.22 (1.01, 1.47)	
No	1.06 (0.95, 1.18)	
Borderline	1.67 (0.88, 3.15)	
Coronary heart disease,(%)		0.575
Yes	1.22 (0.81, 1.84)	
No	1.09 (0.99, 1.19)	
Cancer,(%)		0.895
Yes	1.08 (0.84, 1.39)	
No	1.10 (1.00, 1.22)	

Age, gender, race, BMI, education level, alcohol intake, smoking, diabetes, hypertension, and cancer were adjusted.

PIR, family income to poverty ratio; BMI, body mass index, hs-CRP, high-sensitivity C-reactive protein.

BMI was categorized as <25, 25-29.9, and ≥30 kg/m^2^, corresponding to normal weight, overweight, and obese population, respectively.

**Table 4:** Subgroup analysis of the association between ln transform hs-CRP level and PHQ-9 score.

Subgroup	OR (95% CI)	*P* for interaction
Age		0.014
<60years	0.06 (-0.06, 0.18)	
≥60 years	0.30 (0.15, 0.45)	
Gender		0.009
Male	0.29 (0.15, 0.42)	
Female	0.04 (-0.10, 0.17)	
BMI		0.502
<25 kg/m^2^	0.08 (-0.10, 0.26)	
25-29.9 kg/m^2^	0.17 (0.00, 0.33)	
≥30 kg/m^2^	0.21 (0.07, 0.36)	
Marital status,(%)		0.104
Married/Living with Partner	0.19 (0.06, 0.31)	
Widowed/Divorced/Separated	0.24 (0.05, 0.44)	
Never married	-0.05 (-0.26, 0.17)	
Hypertension(%)		0.237
Yes	0.12 (-0.01, 0.24)	
No	0.23 (0.08, 0.38)	
Diabetes(%)		0.062
Yes	0.37 (0.15, 0.59)	
No	0.12 (0.01, 0.22)	
Borderline	0.51 (-0.06, 1.08)	
Coronary heart disease,(%)		0.014
Yes	0.68 (0.26, 1.11)	
No	0.14 (0.04, 0.23)	
Cancer,(%)		0.471
Yes	0.26 (-0.01, 0.53)	
No	0.15 (0.05, 0.26)	

Age, gender, race, BMI, education level, alcohol intake, smoking, diabetes, hypertension, and cancer were adjusted.

PIR, family income to poverty ratio; BMI, body mass index, PHQ, Patient Health Questionnaire, Hs-CRP, high-sensitivity C-reactive protein.

BMI was categorized as <25, 25-29.9, and ≥30 kg/m^2^, corresponding to normal weight, overweight, and obese population, respectively.

## 4 Discussion

In this cross-sectional study of 6,293 adults, we found that elevated hs-CRP levels may be associated with an increased likelihood of depressive symptoms. This association was similar in subgroups stratified by age, BMI, marital status, hypertension, diabetes, coronary heart disease, and cancer.

To the best of our knowledge, this study is the first to evaluate the correlation between serum hs-CRP levels and depression in US adults. Current evidence implies that inflammation plays a critical role in depression’s pathogenesis due to its ability to trigger the development of the illness. Results from the, 2016 Korean National Health and Nutrition Examination Survey (KNHANES) indicate a potential association between elevated hs-CRP levels and depression in the younger population (12). Furthermore, San Lee et al. previously investigated the relationship between hs-CRP levels in Korean adults and the risk of depression. The results showed that participants with higher hs-CRP were 1.86 times more likely to suffer from depression than the control group. Moreover, the connection between hs-CRP and depression demonstrated biological variances between males and females (11). Previous studies have also reported a correlation between depression and other inflammatory markers. Increased levels of CRP were linked to a higher incidence of depression (11, 20–24). There are also findings suggesting that serum hs-CRP can predict new-onset major depressive disorder (25). However, the association between peripheral CRP levels and depression remains controversial. Mendelian randomization studies have found that elevated plasma CRP levels did not increase the risk of major depression (26). Similarly, the expression of immune-related genes in patients with major depression was not associated with serum CRP levels (27). In addition to blood-based indicators, the link between inflammation and the risk of depression is evident in both dietary habits and systemic inflammation. The components and quality of the diet can also impact the inflammatory state. Research has shown that diets with higher scores on the Dietary Inflammatory Index (DII) are associated with a greater likelihood of depression occurrence (28). There are also reports indicating that the Systemic Inflammation Index (SII) might contribute to the development of depression (29). The results of this study suggest that elevated hs-CRP levels are associated with a higher probability of depressive symptoms.

The subgroup analyses unveiled a positive association between hs-CRP and the prevalence of depressive symptoms, as well as the PHQ-9 score. However, this association reached statistical significance only in men. This observation aligns with prior studies indicating that gender may impact the relationship with hs-CRP, reporting a more robust association between depression and elevated hs-CRP concentrations in men compared to women (10, 11). Vetter et al. identified a notable association between major depression and hs-CRP in men, even after adjusting for confounding factors such as obesity category, age, and other medications known to influence inflammation (30). Maryam et al. evaluated inflammation levels through serum hs-CRP and observed a correlation between the severity of depression and an elevated inflammatory state (10). However, these findings warrant careful interpretation. Gender differences in the inflammatory response linked to depression and the influence of sex hormones may contribute to the stronger association of depression with serum hs-CRP levels in men compared to women (31, 32). There is still a shortage of robust and rigorous research to elucidate this finding. Therefore, future in-depth studies are warranted to explore the role of gender in the association between hs-CRP and depressive symptoms, along with the potential underlying mechanisms. Moreover, our study identified a consistently positive correlation between hs-CRP and depressive symptoms in subgroups categorized by age, BMI, marital status, hypertension, diabetes mellitus, coronary artery disease, and cancer. Importantly, we found no dependence of the association between hs-CRP and depressive symptoms on age, BMI, marital status, hypertension, diabetes mellitus, coronary artery disease, and cancer (all interaction effects with P > 0.05), suggesting that the positive correlation may be applicable to different population settings.

Inadequate sleep is widely recognized to have various detrimental effects. Numerous epidemiological studies have consistently observed the association between sleep patterns and mental health disorders (33). Numerous studies have indicated that sleep disorders constitute a significant risk factor for depression (34, 35). Physical activity has been reported to impact both the physiology and psychology of individuals and is regarded as an effective behavioral intervention for depression (36). A cohort study revealed that physical activity decreased depressive symptoms (37). Physical activity has been identified as a protective factor for hippocampal astrocytes, preventing damage to the central nervous system and reducing the risk of depression (38). Moreover, several chronic medical conditions, such as coronary heart disease (39) and cancer (40), have been recognized for their association with hs-CRP levels. Consequently, lifestyle factors, including smoking, alcohol consumption, physical activity, sleep disorders, as well as medical comorbidities like coronary heart disease and cancer, were taken into account as potential confounding factors in this study.

The correlation between hs-CRP and depression lacks clarity. Hs-CRP mainly investigates low-level inflammation and pre-emptively cautions against inflammatory ailments (41, 42). Current evidence successfully supports the notion that hs-CRP can act as a mediator for inflammatory responses (43–46). Immune dysregulation and overactivation of the immune system are probably responsible for the pathogenesis of depression, and inflammatory cytokines are thought to induce classical depressive symptoms (47, 48). Studies have shown that inflammatory diseases could trigger clinical depression and that elevated inflammation levels potentially increase the risk of new-onset depression (49). Additionally, the remission of clinical depression is often accompanied with normalization of inflammatory markers and depression can instigate the inflammatory reaction in the body (50). Research has indicated that clinical depression is often preceded by psychosocial stress or stressful stimuli. These stressors may augment the production and expression of pro-inflammatory cytokines, resulting in alterations in peripheral immune cells, inflammatory responses, and neurobehavior (49). Additionally, neural responses to stressful stimuli can stimulate inflammatory processes (51). Inflammatory signaling pathways interact with the brain through complex direct or indirect pathways, including neuron-mediated and neuroendocrine-mediated (52–54). Clinical studies constitute a vital foundation for elucidating the link between inflammatory cytokines and depression. Numerous prior investigations have proposed that heightened peripheral CRP levels may augment the risk of depression and serve as predictors for depressive symptoms (55). It has also been found that depressed patients with elevated levels of pro-inflammatory cytokines such as CRP, TNF-α, and IL-6 exhibit suboptimal responses to traditional antidepressant therapies (56, 57). A study conducted by Yayun et al. explored the serum levels of 37 cytokines in individuals with depression. The findings indicated that certain serum factors, including IL-6, IL-8, TNF-α, and TNF-β, could potentially serve as diagnostic biomarkers, allowing differentiation between depressed patients and healthy participants (58). This discovery expands the array of serum cytokine-based biomarkers available for diagnosing depression. Additionally, there is mounting evidence suggesting that many antidepressant medications possess anti-inflammatory properties. The literature documents the efficacy and safety of anti-inflammatory medications, either as monotherapy or adjunctive therapy, for the treatment of depression (59, 60). Nevertheless, the mechanisms through which antidepressants regulate the levels of inflammatory markers are intricate and remain unelucidated. The modulation of inflammatory factors by antidepressants involves neurotransmitter metabolism, the influence of the HPA axis, and the modulation of neurological function (61). In conclusion, depression is a very pleomorphic and heterogeneous phenotype. Although numerous studies have demonstrated the bidirectional pathways between depression and inflammation play a vital role in the pathogenesis of depression and suggest peripheral inflammatory factors may be one of the adjunctive diagnostic methods, most of the literature is based on cross-sectional study designs that lack adequate control for potential confounders. Therefore, population-level prevention, management, and treatment of depression are crucial as a clear goal that we all share. Research suggests that future neuroimaging applications alongside peripheral plasma and cerebrospinal fluid cytokine profiles may offer a more sensitive and specific diagnostic tool for elucidating the pathogenesis of depression (55). However, a comprehensive understanding will necessitate more extensive and in-depth studies.

Our study possesses several strengths. Firstly, it is based on a large dataset from NHANES, enhancing both the reliability and representativeness of our study. Secondly, we have adjusted for several confounding factors, ensuring the stability of our study results and making them relevant to a more extensive population. Nonetheless, the limitations of our study must be addressed. Firstly, previous studies have investigated the association between hs-CRP levels and depressive symptoms in young individuals (12). In contrast to prior research, our study focuses on adults. Due to the design of the NHANES study, individuals under the age of 18 did not participate in the PHQ-9 measure, and data on specific crucial covariates in this study were missing in the individuals under the age of 20. As shown in **Supplementary Table S1**, variability exists between studies investigating the association between hs-CRP levels and depressive symptoms in young people and adults. Consequently, we could not analyze this association for a wider age group. Secondly, owing to the cross-sectional design of the analysis, our study could not indicate a causal relationship between hs-CRP and depression. While the PHQ-9 is a high-sensitivity and specificity screening tool, it is not a clinical diagnostic tool. As a result, our study only confirmed the association between hs-CRP levels and depressive symptoms and could not establish a relationship with depression. Thirdly, it is essential to note that hs-CRP is a non-specific biomarker of acute inflammation, whereas the present study only measured participants’ experiences of depressive symptoms over the past two weeks using PHQ-9. Therefore, it is not appropriate to assume that depressive symptoms equate to depression nor to infer that depression is a chronic pro-inflammatory state. Moreover, the accuracy of the included covariates in this study may not be absolute, potentially influencing result precision. Furthermore, no study has demonstrated the ability of inflammatory factor levels, such as peripheral hs-CRP, to respond to changes in the central nervous system. The sequential and causal relationship between elevated levels of inflammatory factors and the occurrence of depressive symptoms cannot be entirely determined. Hence, our findings may not completely reflect the actual situation. The detailed molecular mechanisms supporting our findings remain uncertain, and additional laboratory-based experiments are required in the future.

The present findings underscore the potential significance of hs-CRP levels in predicting the risk of depressive symptoms. However, further extensive prospective studies are necessary to validate our results and clarify this association’s precise causality. This may include matched-design studies to investigate whether elevated peripheral hs-CRP levels contribute to the risk of depression onset or recurrence. Additionally, exploring whether treatment with anti-inflammatory therapy can lower peripheral hs-CRP levels and alleviate patients’ depressive symptoms and whether antidepressant treatment alone can reduce peripheral hs-CRP levels requires further investigation. Subsequent studies are essential to substantiate these hypotheses.

## 5 Conclusion

In conclusion, our study findings indicate a positive association between hs-CRP and depressive symptoms, with this association being more pronounced in the male population. We hypothesized that timely management and control of hs-CRP levels might improve or reduce depressive symptoms. However, further large-scale prospective studies are still required to elucidate the precise causal relationship.

## Data availability statement

The original contributions presented in the study are included in the article/**Supplementary Material**, further inquiries can be directed to the corresponding author/s.

## Ethics statement

The studies involving humans were approved by NCHS Ethics Review Board (ERB). The studies were conducted in accordance with the local legislation and institutional requirements. Written informed consent for participation in this study was provided by the participants’ legal guardians/next of kin.

## Author contributions

YJ: Conceptualization, Data curation, Investigation, Methodology, Software, Visualization, Writing – original draft, Writing – review & editing. JW: Funding acquisition, Project administration, Resources, Writing – review & editing. HC: Data curation, Writing – original draft, Investigation. JL: Data curation, Methodology, Writing – original draft. MC: Writing – review & editing, Data curation, Methodology.
