# Supplementary material for: Association between hs-CRP and depressive symptoms: a cross-sectional study
Source: Front Psychiatry. 2024 Mar 26;15:1339208. doi: 10.3389/fpsyt.2024.1339208 (PMC11002220; doi:10.3389/fpsyt.2024.1339208)
Supplement: Supplementary file 1 [file Table_1.docx]

| **Characteristics** | **Excluded participants** | **Included participants** | ***P*-value*** |
| --- | --- | --- | --- |
|  | **N=9,267** | **N=6,293** |  |
| Age,(year) | 13.00 (5.00-33.00) | 52.00 (36.00-64.00) | <0.001 |
| Gender,(%) |  |  | 0.035 |
| Male | 50.32 | 48.59 |  |
| Female | 49.68 | 51.41 |  |
| Race,(%) |  |  | <0.001 |
| Mexican American | 13.64 | 11.54 |  |
| Other Hispanic | 9.99 | 9.82 |  |
| Non-Hispanic White | 31.19 | 37.84 |  |
| Non-Hispanic Black | 27.45 | 24.69 |  |
| Other Races | 17.73 | 16.11 |  |
| Education level,(%)) |  |  | <0.001 |
| Less than high school | 24.57 | 16.49 |  |
| High school or GED | 24.63 | 23.85 |  |
| Above high school | 50.29 | 59.65 |  |
| Smoking,(%) |  |  | <0.001 |
| Yes | 35.50 | 42.62 |  |
| No | 64.35 | 57.38 |  |
| Alcohol consumption,(%) |  |  |  |
| Yes | 83.34 | 91.72 |  |
| No | 16.66 | 8.28 |  |
| Marital status,(%) |  |  | <0.001 |
| Married/Living with Partner | 53.35 | 58.97 |  |
| Widowed/Divorced/Separated | 25.25 | 22.34 |  |
| Never married | 21.06 | 18.69 |  |
| Physical activity,(%) |  |  | <0.001 |
| Yes | 38.09 | 41.46 |  |
| No | 61.85 | 58.54 |  |
| Hypertension,(%) |  |  | <0.001 |
| Yes | 30.75 | 38.09 |  |
| No | 68.94 | 61.91 |  |
| Diabetes,(%) |  |  | <0.001 |
| Yes | 5.59 | 15.24 |  |
| No | 93.18 | 81.82 |  |
| Borderline | 1.14 | 2.94 |  |
| Cancer,(%) |  |  | 0.006 |
| Yes | 11.50 | 10.61 |  |
| No | 88.36 | 89.39 |  |
| Coronary heart disease,(%) |  |  | 0.897 |
| Yes | 4.73 | 4.53 |  |
| No | 94.96 | 95.47 |  |
| Sleep disorders,(%) |  |  | <0.001 |
| Yes | 22.12 | 29.80 |  |
| No | 77.68 | 70.20 |  |
| BMI,(kg/m^2^) | 21.70 (17.10-27.80) | 28.90 (25.00-33.90) | <0.001 |
| PIR | 1.71 (0.87-3.46) | 2.32 (1.21-4.31) | <0.001 |
| hs-CRP level,(mg/L) | 0.75 (0.34-2.36) | 2.00 (0.86-4.47) | <0.001 |
| PHQ-9 score | 2.00 (0.00-5.00) | 2.00 (0.00-5.00) | 0.252 |

**Table S1:** Basic characteristics of excluded and included participants

Median (Q1-Q3) for: continuous variables, (%)for categorical variables.

PIR, family income to poverty ratio; BMI, body mass index; PHQ, Patient Health Questionnaire, hs-CRP, high-sensitivity C-reactive protein.
